# Virologic and immunologic outcomes of treatment with integrase inhibitors in a real-world setting: The RESPOND cohort consortium

**DOI:** 10.1371/journal.pone.0243625

**Published:** 2020-12-31

**Authors:** Bastian Neesgaard, Amanda Mocroft, Robert Zangerle, Ferdinand Wit, Fiona Lampe, Huldrych F. Günthard, Coca Necsoi, Matthew Law, Cristina Mussini, Antonella Castagna, Antonella d’Arminio Monforte, Christian Pradier, Nikoloz Chkhartisvilli, Juliana Reyes-Uruena, Jörg Janne Vehreschild, Jan-Christian Wasmuth, Anders Sönnerborg, Christoph Stephan, Lauren Greenberg, Josep M. Llibre, Alain Volny-Anne, Lars Peters, Annegret Pelchen-Matthews, Vani Vannappagari, Joel Gallant, Armin Rieger, Mike Youle, Dominique Braun, Stephane De Wit, Kathy Petoumenos, Vanni Borghi, Vincenzo Spagnuolo, Tengiz Tsertsvadze, Jens Lundgren, Lene Ryom, for The RESPOND study group

**Affiliations:** 1 CHIP, Department of Infectious Diseases, Rigshospitalet, University of Copenhagen, Copenhagen, Denmark; 2 Centre for Clinical Research, Epidemiology, Modelling and Evaluation (CREME), Institute for Global Health, University College London, London, United Kingdom; 3 Austrian HIV Cohort Study (AHIVCOS), Medizinische Universität Innsbruck, Innsbruck, Austria; 4 AIDS Therapy Evaluation in the Netherlands Cohort (ATHENA), Stichting HIV Monitoring (SHM), Amsterdam, Netherlands; 5 Royal Free Hospital, University College London, London, United Kingdom; 6 Division of Infectious Diseases and Hospital Epidemiology, University Hospital Zurich, Zurich, Switzerland; 7 Institute of Medical Virology, University of Zurich, Zurich, Switzerland; 8 CHU Saint-Pierre, Centre de Recherche en Maladies Infectieuses a.s.b.l., Brussels, Belgium; 9 The Australian HIV Observational Database (AHOD), UNSW, Sydney, Australia; 10 Modena HIV Cohort, Università degli Studi di Modena, Modena, Italy; 11 San Raffaele Scientific Institute, Università Vita-Salute San Raffaele, Milano, Italy; 12 Italian Cohort Naive Antiretrovirals (ICONA), ASST Santi Paolo e Carlo, Milano, Italy; 13 Nice HIV Cohort, Université Côte d’Azur et Centre Hospitalier Universitaire, Nice, France; 14 Infectious Diseases, AIDS and Clinical Immunology Research Center, Tbilisi, Georgia; 15 PISCIS Cohort, Centre d’Estudis Epidemiològics sobre les Infeccions de Transmissió Sexual i Sida de Catalunya (CEEISCAT), CIBERESP, Badalona, Spain; 16 Medical Department 2, Hematology/Oncology, University Hospital of Frankfurt, Frankfurt, Germany; 17 Department I for Internal Medicine, University Hospital of Cologne, Cologne, Germany; 18 University Hospital Bonn, Bonn, Germany; 19 Swedish InfCare HIV Cohort, Karolinska University Hospital, Stockholm, Sweden; 20 Infectious Diseases Unit, Medical Dept. no.2, Frankfurt University Hospital, Goethe-University, Frankfurt, Germany; 21 Infectious Diseases and Fight AIDS Foundation, Hospital Universitari Germans Trias i Pujol, Barcelona, Spain; 22 European AIDS Treatment Group (EATG), Brussels, Belgium; 23 ViiV Healthcare, Research Triangle, North Carolina, United States of America; 24 Gilead Sciences, Foster City, California, United States of America; 25 Wiener Medizinische Universität, Vienna, Austria; University of Cape Town, SOUTH AFRICA

## Abstract

**Objectives:**

To compare virologic and immunologic outcomes of integrase inhibitor (INSTI)-containing, contemporary boosted protease inhibitor (PI/b)-containing and non-nucleotide reverse transcriptase inhibitor (NNRTI)-containing regimens in a real-life setting.

**Methods:**

Using logistic regression, virologic and immunologic outcomes of INSTI use were compared to outcomes of PI/b or NNRTI treatment 12 months after treatment start or switch, for participants in the RESPOND cohort consortium. A composite treatment outcome (cTO) was used, defining success as viral load (VL) <200 copies/mL and failure as at least one of: VL ≥200 copies/mL, unknown VL in the time window, any changes of antiretroviral therapy (ART) regimen, AIDS, or death. In addition, on-treatment analysis including only individuals with known VL and no regimen changes was performed. Favorable immunologic response was defined as a 25% increase in CD4 count or as reaching ≥750 CD4 cells/μL.

**Results:**

Between January 2012 and January 2019, 13,703 (33.0% ART-naïve) individuals were included, of whom 7,147 started/switched to a regimen with an INSTI, 3,102 to a PI/b and 3,454 to an NNRTI-containing regimen. The main reason for cTO failure in all treatment groups were changes in ART regimen. Compared to INSTIs, the adjusted odds ratio (aOR) of cTO success was significantly lower for PI/b (0.74 [95% confidence interval, CI 0.67–0.82], p <0.001), but similar for NNRTIs (1.07 [CI 0.97–1.17], p = 0.11). On-treatment analysis and sensitivity analyses using a VL cut-off of 50 copies/mL were consistent. Compared to INSTIs, the aORs of a 25% increase in CD4 count were lower for NNRTIs (0.80 [CI 0.71–0.91], p<0.001) and PI/b (0.87 [CI 0.76–0.99], p = 0.04).

**Conclusion:**

In this large analysis of a real-world population, cTO and on-treatment success were similar between INSTIs and NNRTIs, but lower for PI/b, though residual confounding cannot be fully excluded. Obtaining favorable immunologic outcomes were more likely for INSTIs than the other drug classes.

## Introduction

Integrase inhibitors (INSTIs) have excelled in randomized clinical trials (RCTs) by showing potent and swift suppression of human immunodeficiency virus (HIV), good short-term drug safety and tolerability, and, for second generation INSTIs, high genetic barriers to resistance [1–7]. In turn, this has led to INSTI-based regimens being recommended as first-line antiretroviral therapy (ART) by European, North American and WHO guidelines, above or alongside contemporary ritonavir- or cobicistat-boosted protease inhibitor (PI/b)-containing and non-nucleotide reverse transcriptase inhibitor (NNRTI)-containing regimens [8–12]. However, RCTs typically enroll only a modestly sized and selected study population, where females, individuals with high HIV viral loads (VLs), low CD4 counts, comorbidities or coinfections are commonly underrepresented. Results from RCTs should therefore be complemented by investigations from large, heterogeneous, real-life, observational studies, ensuring that findings from RCTs are generalizable to the majority of people living with HIV (PLWH).

In this study we analysed virologic and immunologic outcomes of INSTI-containing regimens compared to contemporary PI/b- and NNRTI-containing regimens within the large heterogenous RESPOND cohort consortium.

## Methods

### Study design

The RESPOND cohort consortium (https://chip.dk/Studies/RESPOND) was initiated in 2017 as an international collaboration between 17 pre-existing, prospective European and Australian based cohorts of HIV-1 positive participants (see S1 Appendix for RESPOND inclusion/exclusion criteria). At present, the RESPOND consortium follows >29,000 PLWH.

The Outcomes Study under the consortium was formed to investigate use and outcomes of treatment with contemporary ART [13]. Information systematically collected on all participants includes demographics (e.g. age, sex and ethnicity), HIV related variables (e.g. VL, CD4 counts, route of HIV acquisition and AIDS events), detailed information on ART use and reasons for discontinuation, laboratory measures (e.g. creatinine, liver transferases and glucose), comorbidities (e.g. diabetes, hypertension and viral hepatitis coinfections) [14]. In addition, incident clinical events including cancer, cardiovascular, renal and liver disease are reported on designated forms and centrally validated. All collected data is transferred electronically to RESPOND, and undergoes extensive quality assessments [14].

### Ethics

All studies carried out within the RESPOND consortium are conducted according to the Declaration of Helsinki and the requirements of Good Clinical Practice (GCP) as defined in the European Union’s (EU) GCP Directive. All data supplied to RESPOND follows local or national guidelines as appropriate, and enrolled participants are pseudonymized by assignment of a unique identifier, by the participating cohort before data transfer. As data controller, the Coordinating Centre (CC) located within the Danish Capital Region of Copenhagen, Denmark, stores, shares and protects data in accordance with current legislation and under approval by The Danish Data Protection Agency (j.nr.: RH-2018-15, 26/1/2018), currently under the EU’s General Data Protection Regulation (EU) 2016/679.

RESPOND is registered at Clinicaltrials.gov (Identifier: NCT04090151).

### Inclusion criteria

For this specific analysis, we included ART-naïve and -experienced individuals, aged over 18 years who started or switched to an INSTI, PI/b or NNRTI-containing regimen with at least three antiretroviral drugs (ARVs). The eligible regimen was started between January 2012 and January 2019, and all included had a CD4 count and VL measured in the 12 months before or 3 months after treatment start/switch (baseline). Treatment-experienced individuals were naïve to the specific third agent they started when eligible for inclusion (e.g. an individual switching to darunavir had not previously been treated with darunavir).

### Definition of outcomes

All outcomes were assessed 12 months after baseline. The VL and CD4 count closest to this time were used, allowing a maximum time window of 3 months to either side (12±3 months).

To estimate the durability of regimens, a composite treatment outcome (cTO) was used, defining treatment success as VL <200 copies(cp)/mL and failure as occurrence of at least one of either: VL ≥200 copies (cp)/mL, unknown VL in the time window, any ART regimen change, AIDS event, or death. Switching from a multi-tablet combination regimen to a single-tablet combination regimen including the same agents, or vice versa, was not considered as a regimen change. Conversely, switch to a two-drug regimen and/or any changes in individual regimen components were viewed as regimen changes (e.g. if tenofovir disoproxil fumarate [TDF] was changed to tenofovir alafenamide [TAF], it was regarded as a treatment change, as it could be related to drug toxicity).

Sensitivity analyses tested the robustness of the cTO by excluding either individuals with unknown VL values within the time widow or those with any regimen change. Furthermore, an analysis which did not define changes in the NRTI backbone as a regimen change was performed.

Additionally, the efficacy of the regimens to control HIV (VL <200 cp/mL) was estimated using on-treatment analysis, including only individuals with a known VL at 12±3 months and no regimen changes within the period.

The use of a VL <200 cp/mL threshold for the cTO and on-treatment analysis followed a consensus agreement within the RESPOND virologic outcomes working group, reflecting differences in the sensitivity of VL assays available in different cohorts. Sensitivity analyses using a VL <50 cp/mL threshold were also performed.

A favorable immunologic response was defined as a 25% increase in CD4 count from baseline. As individuals with high CD4 counts at baseline would presumably have lower odds of reaching such an increase, we also defined a favorable immunologic response as reaching CD4 count ≥750 cells/μL (excluding those with CD4 count ≥750 cells/μL at baseline), following the rationale described in earlier work by Mocroft et al. [15].

To ascertain if there were any variations in outcomes depending on treatment status at baseline, outcomes were determined for three pre-specified sub-groups: ART-naïve individuals, ART-experienced individuals switching to a new ART-regimen with ongoing viremia (≥200 cp/mL), and ART-experienced individuals switching to a new ART-regimen with virologic control (<200 cp/mL). Given the heterogeneity of the populations, tests for interactions between ART-experience and treatment status at baseline for each outcome were planned a priori.

### Statistics

Descriptive statistics were summarized as frequencies and proportions with χ^2^ P-values for categorical variables. For continuous variables, data were presented as medians and interquartile ranges (IQR), with P-values from the Wilcoxon-Mann-Whitney test.

Virologic and immunologic outcomes for individuals starting/switching to an INSTI (dolutegravir, raltegravir or cobicistat-boosted elvitegravir)-containing regimen were compared to participants starting/switching to a PI/b (ritonavir- or cobicistat-boosted darunavir or atazanavir)-containing or NNRTI (rilpivirine or efavirenz)-containing regimen, using separate logistic regression models.

Factors considered for multivariable analyses included demographics (age, sex, and ethnicity) and region of origin, categorized as West Central Europe (Austria, Belgium, France, Germany, Luxembourg, Switzerland), South Europe and Argentina (Argentina, Greece, Israel, Italy, Portugal, Spain), North Europe and Australia (Australia, Denmark, Norway, Sweden, Finland, Iceland, Ireland, Netherlands, United Kingdom; Australia was included here based on the small number of individuals, and similarities with the United Kingdom), East Central Europe (Bosnia-Herzegovina, Croatia, Czech Republic, Hungary, Poland, Romania, Serbia, Slovenia, Slovakia) and East Europe (Belarus, Estonia, Georgia, Latvia, Lithuania, Russia, Ukraine). Other variables included HIV-related factors (VL, CD4 count, route of HIV acquisition, ART, and prior AIDS), hepatitis B and C coinfection, cardiovascular risk factors (smoking status, hypertension and diabetes) and other non-AIDS events (chronic kidney disease, end stage liver-and renal disease, cardiovascular disease, fractures and malignancies). All factors included in the models were decided a priori. Full lists of variables included in the specific multivariate models are shown in the figure legends.

As some cohorts did not have complete data on comorbidities, it was not possible to adjust for both cohort and comorbidities in the same analyses, due to the risk of collinearity. Therefore, our main analyses focused on adjusting for comorbidities as listed above, while acknowledging that there was some missing data. Sensitivity analyses adjusting for cohort rather than comorbidities were also performed.

Finally, sensitivity analyses restricted to ART-naïve individuals starting treatment after 16^th^ January 2014 (the date dolutegravir was licensed in Europe) were performed.

All analyses were performed using SAS (Statistical Analysis Software, Cary, NC, US) version 9.4., all tests of significance were 2-sided with p<0.05 used for statistical significance and all confidence intervals (CI) were 95%.

## Results

### Baseline demographics and clinical characteristics

In total, 13,703 participants were included (Table 1). Of these, 4,967 (52.2%) were treated with an INSTI (dolutegravir 3,839, raltegravir 1,738 and elvitegravir 1,570), 3,102 (22.6%) with a PI/b (darunavir/b 2,381, atazanavir/b 721), and 3,454 (25.9%) with an NNRTI-containing regimen (rilpivirine 2,508, efavirenz 946). The most common nucleos(t)ide reverse transcriptase inhibitor (NRTI) backbone used in all three treatment groups was TDF/emtricitabine (FTC), followed by abacavir (ABC)/lamivudine (3TC) in the INSTI and PI/b groups and TAF/FTC in the NNRTI group. Median time since initiation of first ARV was longest for individuals in the INSTI group (13 years [IQR 7–18]) followed by individuals in the PI/b and NNRTI groups (10 [5–16] and 8 [4–14], respectively). Likewise, the median number of ARVs previously exposed to, was slightly higher in the INSTI group (6 [4–9]) than in the PI/b and NNRTI groups (5 [3–8] and 5 [3–7], respectively). At baseline, ART-naïve individuals constituted 26.8%, 40.2% and 41.7% of the INSTI, PI/b and NNRTI groups, respectively. There was a greater proportion of ART-experienced individuals with VL <200 cp/mL in the INSTI group compared to the PI/b and NNRTI groups (65.7% vs. 43.6% and 55.6%, respectively). Baseline characteristics stratified by treatment status at baseline are provided in S1 Table.

**Table 1:** Baseline demographics and clinical characteristics of study participants, stratified by treatment group.

		All		INSTI		PI/b		NNRTI	
		n	%	n	%	n	%	n	%
		13703	100.0	7147	52.2	3102	22.6	3454	25.2
Sex	Female	3390	24.7	1693	23.7	936	30.2	761	22.0
	Male	10313	75.3	5454	76.3	2166	69.8	2693	78.0
Ethnicity	White	9625	70.2	5049	70.6	2061	66.4	2515	72.8
	Other	2162	15.8	994	13.9	638	20.6	530	15.3
	Unknown	1916	14.0	1104	15.4	403	13.0	409	11.8
Region	South Europe and Argentina	3825	27.9	1752	24.5	943	30.4	1130	32.7
	West Central Europe	7364	53.7	4121	57.7	1608	51.8	1635	47.3
	North Europe and Australia	1489	10.9	885	12.4	296	9.5	308	8.9
	Central East Europe	623	4.5	325	4.5	143	4,6	155	4.5
	East Europe	402	2.9	64	0.9	112	3.6	226	6.5
Route of HIV acquisition	MSM	6322	46.1	3377	47.3	1194	38.5	1751	50.7
	IDU	1768	12.9	998	14.0	460	14.8	310	9.0
	Heterosexual	4626	33.8	2213	31.0	1216	39.2	1197	34.7
	Other/Unknown	987	7.2	559	7.8	232	7.5	196	5.7
Treatment experience/viral load (cp/mL)	ART-naïve	4521	33.0	1914	26.8	1248	40.2	1359	39.3
	ART-experienced, VL ≥200 cp/mL	1213	8.9	538	7.5	500	16.1	175	5.1
	ART-experienced, VL <200 cp/mL	7969	58.2	4695	65.7	1354	43.6	1920	55.6
NRTI Backbone	TDF/FTC	8158	59.5	3318	46.4	2139	69.0	2701	78.2
	TAF/FTC	1116	8.1	727	10.2	33	1,1	356	10.3
	ABC/3TC	3720	27,1	2692	37,7	722	23,3	306	8,9
	Other	709	5.2	410	5.7	208	6,7	91	2.6
Prior AIDS	Yes	2741	20.0	1606	22.5	640	20.6	495	14.3
Hepatitis B	Positive	633	4.6	342	4.8	133	4.3	158	4.6
Hepatitis C	Positive	2731	19.9	1598	22.4	628	20.2	505	14.6
BMI (kg/m^2^)	≤18	332	2.4	189	2.6	92	3.0	51	1.5
	18–25	6143	44.8	3441	48.1	1282	41.3	1420	41.1
	25–30	2716	19.8	1592	22.3	493	15.9	631	18.3
	>30	877	6.4	499	7.0	170	5.5	208	6.0
Smoking	Current	4241	30.9	2273	31.8	925	29.8	1043	30.2
Hypertension	Yes	3402	24.8	2108	29.5	574	18.5	720	20.8
Diabetes	Yes	731	5.3	482	6.7	109	3.5	140	4.1
Cardiovascular disease	Yes	386	2.8	261	3.7	62	2.0	63	1.8
Chronic kidney disease	Yes	400	2.9	295	4.1	66	2.1	39	1.1
**Continuous variables**		**Median**	**(IQR)**	**Median**	**(IQR)**	**Median**	**(IQR)**	**Median**	**(IQR)**
Age (years)		46	37–53	48	39–54	43	35–51	43	36–51
Baseline CD4 (cells/μL)		510	328–724	551	359–761	411	230–624	510	356–710
Nadir CD4 (cells/μL)		228	105–358	216	99–346	200	80–330	273	156–390
ARV drugs previous taken*(n)		6	3–8	6	4–9	5	3–8	5	3–7
Years since initiation of first ARV (years)		11	6–17	13	7–18	10	5–16	8	4–14
Baseline date (mm/yy)		10/14	07/13–08/16	06/14	10/14–04/16	07/13	07/12–09/14	06/13	03/12–08/14

All percentages are column percentages

The proportion of individuals with unknown/missing data were (%): Mode of transmission 6.8; ethnicity 11.8; hepatitis B 16.8; hepatitis C 13.8; BMI 29.5; smoking status 39.0; hypertension 28.9; diabetes 8.5; cardiovascular disease 26.3; chronic kidney disease 19.2.

Regions: WestCentral Europe: Austria, Belgium, France, Germany, Luxembourg, Switzerland; South Europe and Argentina: Argentina, Greece, Israel, Italy, Portugal, Spain; North Europe and Australia: Australia, Denmark, Norway, Sweden, Finland, Iceland, Ireland, Netherlands, United Kingdom; East Central Europe: Bosnia-Herzegovina, Croatia, Czech Republic, Hungary, Poland, Romania, Serbia, Slovenia, Slovakia; Eastern Europe: Belarus, Estonia, Georgia, Latvia, Lithuania, Russia, Ukraine.

Hypertension: Defined as use of antihypertensive drugs, systolic blood pressure >140 mmHg and/or diastolic blood pressure >90 mmHg.

Diabetes: A clinical diagnosis of diabetes, use of antidiabetic drugs and/or blood glucose measurement ≥11.1 mmol/l or HbA1C ≥48 mmol/mol.

HCV Positive: Positive if ever had a positive HCV antibody test, HCV RNA test, HCV genotype, or received HCV treatment prior to baseline. HBV positive: Hepatitis B surface antigen positive

Chronic kidney disease: Defined as confirmed (>3 months apart) eGFR ≤ 60 ml/min/1.73 m^2^, calculated by the CKD-EPI formula.

Cardiovascular disease includes prior myocardial infarction, stroke or invasive cardiovascular procedure.

*Among ART-experienced

Abbreviations: 3TC, lamivudine; ABC, abacavir; ART, anti-retroviral therapy; ARV, anti-retroviral drug; BMI, body-mass index; cp, copies of RNA; FTC, emtricitabine; IDU, intravenous drug user; MSM, men who have sex with men; NRTI, nucleos(t)ide reverse transcriptase inhibitor; TDF: tenofovir disoproxil fumarate; TAF: tenofovir alafenamide; VL, viral load.

All p-values for comparison of baseline characteristics between treatment groups were < 0,001.

Overall, 75.3% of those included were of male sex, with men who have sex with men (MSM) being the most common route of HIV acquisition (46.1%; Table 1). The most common region of origin was West Central Europe (53.7%), followed by South Europe and Argentina (27.9%) and North Europe and Australia (10.9%). Median age was slightly higher in the INSTI group than in the PI/b or NNRTI groups (48 [IQR 39–54] years vs. 43 [35–51] and 43 [36–51] years, respectively). Median CD4 count was highest for individuals on INSTIs in comparison with individuals on PI/b or NNRTIs (551 [IQR 359–761] cells/μL vs. 411 [230–624] or 510 [356–710] cells/μL, respectively). Furthermore, a greater proportion of current smokers, individuals with BMI >25 kg/m^2^, and comorbidities such as hypertension, diabetes, chronic kidney disease and prior cardiovascular disease, was found in the INSTI group compared the other two treatment groups. All p-values for all comparison of baseline characteristics between treatment groups were < 0.001.

### Virologic outcomes

**Fig 1:**
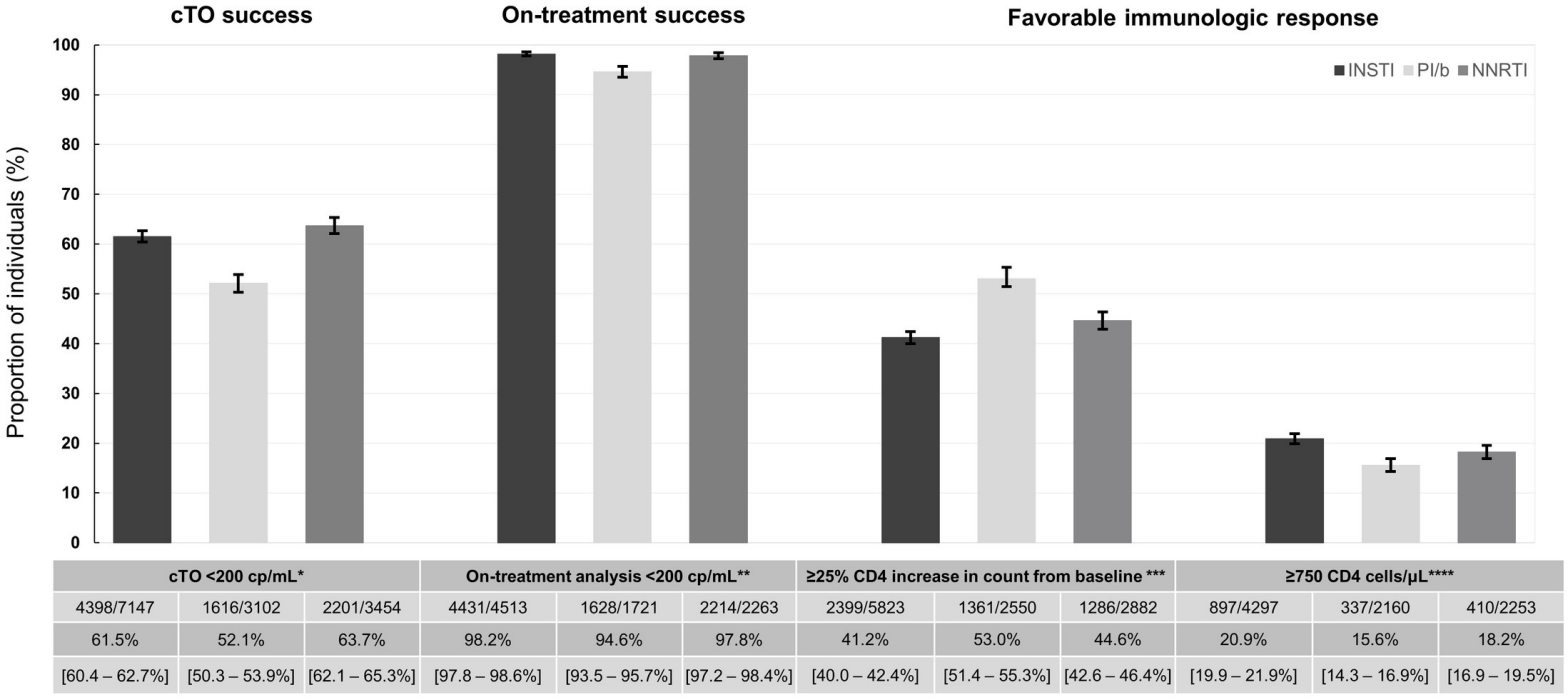
Proportions of individuals with composite treatment outcome (cTO) success, on-treatment success, or favorable immunologic response after 12±3 months. Error bars and numbers in the 3^rd^ row in the table indicate 95% confidence intervals. * cTO failure was defined as ≥1 of VL ≥200 cp/mL, unknown VL, any ART regimen change, AIDS events, or death. ** Individuals with known VL at 12±3 months without ART regimen change in the periods (N = INSTI 4513; PI/b 1721; NNRTI 2263). *** Persons with known CD4 counts at 12±3 months (N = INSTI 5823; PI/b 2550; NNRTI 2882). ****Persons with known CD4 counts at 12±3 months, excluding those with ≥750 CD4 cells/μL at baseline (N = INSTI 4297; PI/b 2160; NNRTI 2253).

**Fig 2:**
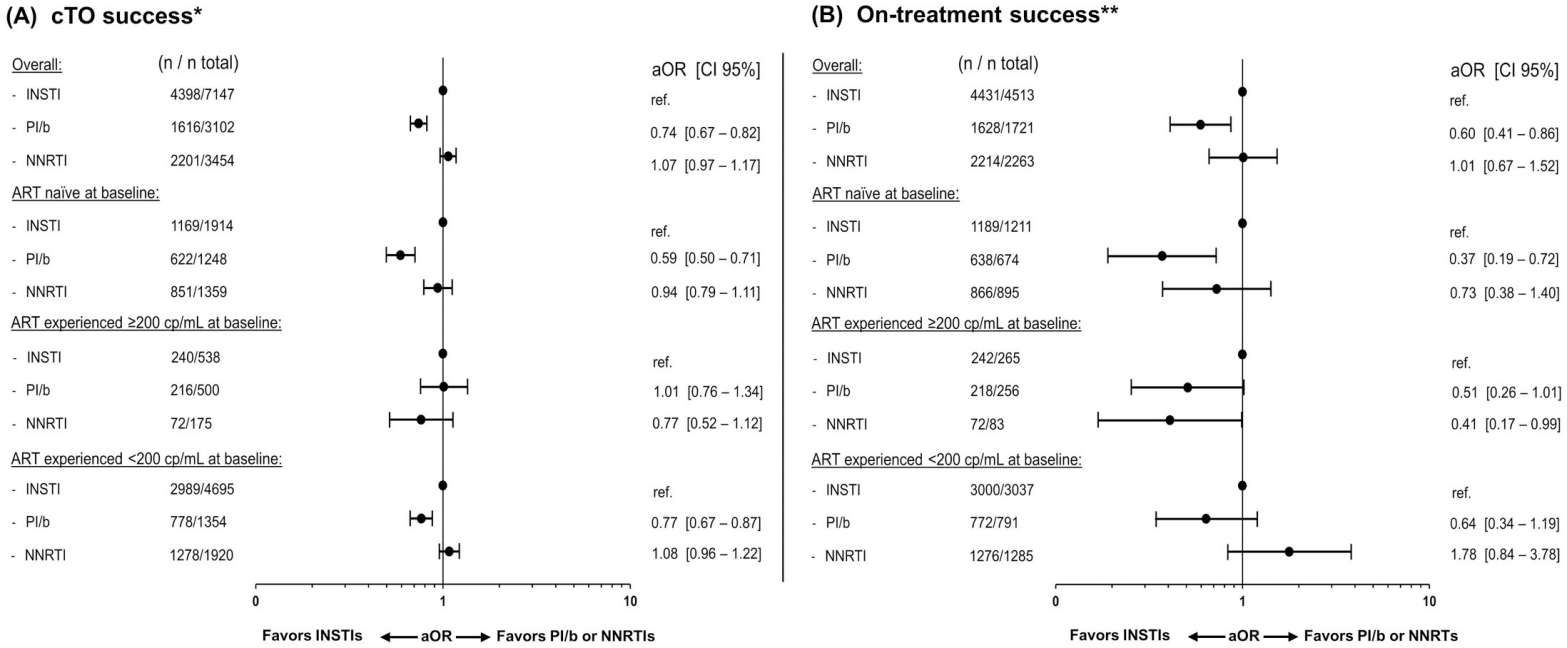
Adjusted odds ratios (aOR) of composite treatment outcome (cTO) success and on-treatment success at 12±3 months. Forest plots showing the aOR of cTO success (A) or on-treatment success (B). A controlled viral load was defined as <200 cp/mL. The multivariable models were adjusted for: Age (per ten years older), ethnicity, mode of transmission, baseline date (per year later), baseline smoking status, hypertension, diabetes, HBV and HCV status, prior AIDS event, cardiovascular disease, chronic kidney disease, end stage liver disease, non-AIDS-defining malignancies and prior fractures, viral load(<200 cp/mL, ≥200 cp/mL at baseline) and treatment status, CD4 count (nadir and baseline; both per 100 cells higher), treatment regimen and number of drugs in regimen. * cTO failure was defined as ≥1 of VL ≥200 cp/mL, unknown VL, any ART regimen change, AIDS events, or death. ** Individuals with known VL at 12±3 months without ART regimen change in the period.

The crude proportion of cTO success was lowest in the PI/b group, while being fairly similar between the INSTI and NNRTI groups (52.1% [95% CI 50.3–53.9] vs. 61.5% [60.4–62.7] and 63.7% [62.1–65.3], p <0.001 respectively; Fig 1). Similar results were seen after adjustments. The adjusted odds ratio (aOR) of cTO success was significantly lower for PI/b compared to INSTIs (aOR 0.74 [CI 0.67–0.82], p <0.001; Fig 2A), while no significant difference was seen when comparing NNRTIs and INSTIs (aOR 1.07 [0.97–1.17], p = 0.11).

**Fig 3:**
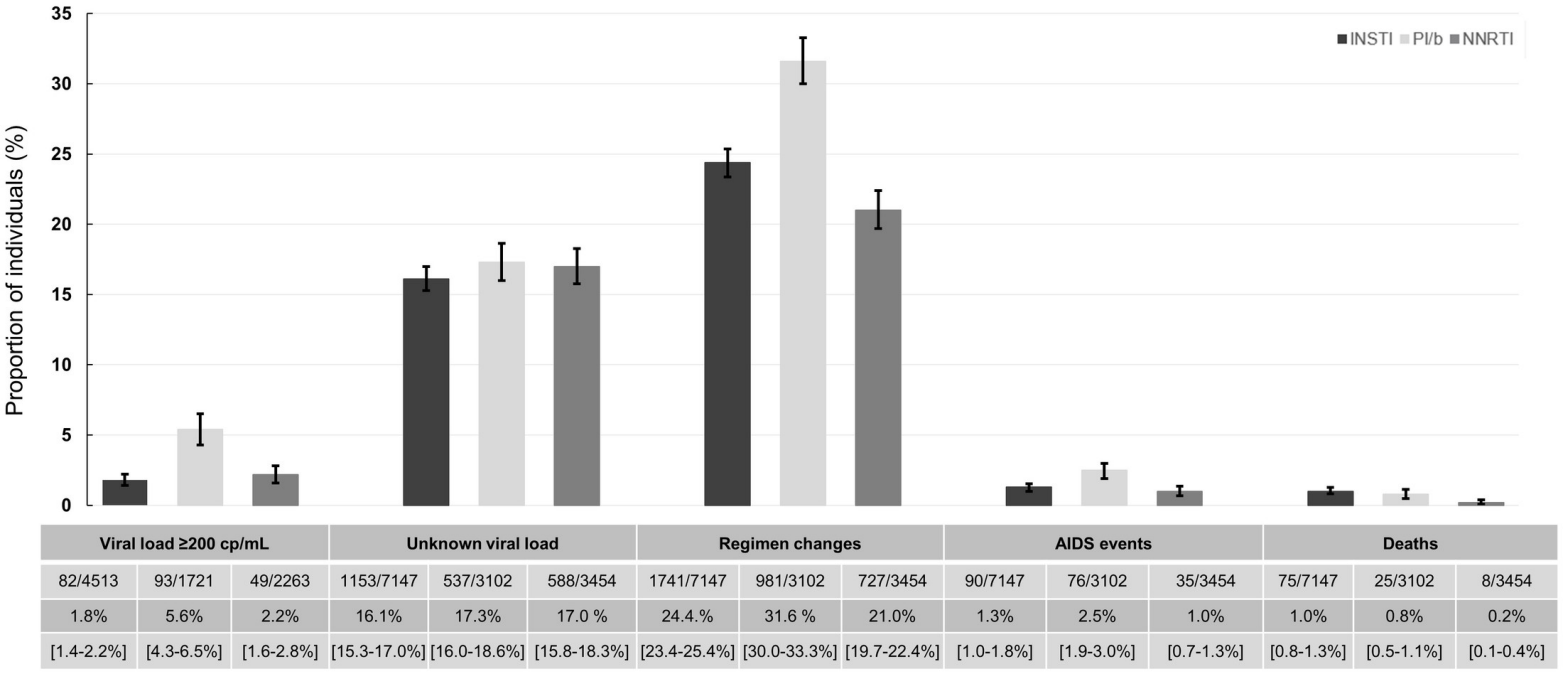
Reasons for cTO failure at 12±3 months. Error bars and 3^rd^ row in the table indicate 95% confidence intervals. Total numbers with cTO failure: INSTI: 2749/7147; PI/b: 1396/3102; NNRTI 1253/3454. *cTO failure: ≥1 of: VL ≥200 cp/mL, unknown VL, any antiretroviral treatment (ART)-regimen change, AIDS, or death (note it was possible to fail more than one parameter).

The main reasons for cTO failure in all three treatment groups were regimen changes and unknown VL within the time window (Fig 3). However, while proportions of individuals with unknown VLs were similar between groups (16.1% [CI 15.3–17.0], 17.3% [16.0–18.6] and 17.0% [15.8–18.3], p = 0.26, for INSTIs, PI/b and NNRTIs respectively), regimen changes occurred more often in the PI/b group, compared to the INSTI and NNRTI groups (31.6% [30.0–33.3] vs. 24.4% [23.4–25.4] and 21.0% [19.7–22.4], p <0.001 respectively). In addition, the number of individuals experiencing virologic failure in the PI/b group was higher in comparison with the INSTI and NNRTI group (5.4% [4.3–6.5] vs. 1.8% [1.4–2.2] and 2.2% [1.6–2.8], p <0.001, respectively). Overall, the number of individuals developing AIDS (n = 201) or who died during follow-up (n = 108) was low. However, there were some differences between the groups. The proportion with AIDS was slightly higher in the PI/b group compared to the INSTI and NNRTI group (2.5% [1.9–3.0] vs 1.3% [1.0–1.5] and 1.0% [0.7–1.3]; p<0.0001 for comparison). The proportion of deaths was highest in the INSTI group and lowest in the NNRTI group (1.0% [0.8–1.3], 0.8% [0.5–1.3] and 0.2% [0.1–0.4]; p< 0.0001).

Overall, 8,497/13,703 individuals (62.0%) were included in the on-treatment analyses (53.1% on an INSTI, 20.3% on a PI/b and 26.6% on a NNRTI). The crude proportion of individuals achieving success was higher in the INSTI and NNRTI groups than in the PI/b group (98.2% [CI 97.8–98.6] and 97.8% [97.2–98.4], respectively vs. 94.6% [93.5–95.7], p <0.001; Fig 1). Correspondingly, the aOR of on-treatment success was similar for the NNRTI group when compared to the INSTI group (1.01 [0.67–1.52], p = 0.97), but significantly lower for the PI/b group (0.60 [0.41–0.86], p = 0.01); Fig 2B).

### Immunologic outcomes

**Fig 4:**
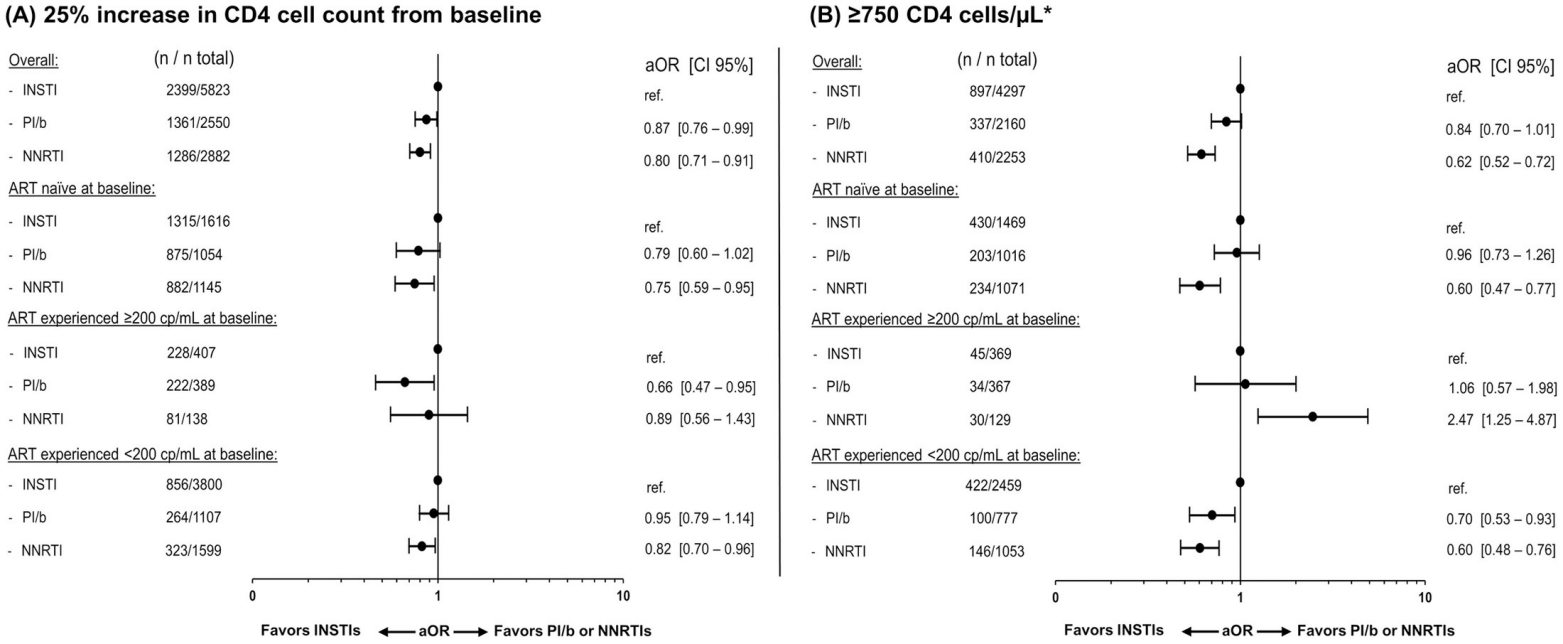
Adjusted odds ratios (aOR) of a favorable immunologic response at 12±3 months. Immunologic response was defined as a 25% increase in CD4 cell counts (A) or reaching a CD4 cell count ≥750 cells/μL (B). Models were adjusted for age (per ten years older), ethnicity, mode of transmission, baseline date (per year later), baseline smoking status, hypertension, diabetes, HBV and HCV status, prior AIDS event, cardiovascular disease, chronic kidney disease, end stage liver disease, non-AIDS-defining malignancies and prior fractures, viral load (<200 cp/mL, ≥200 cp/mL at baseline) and treatment status, CD4 count (nadir and baseline; both per 100 cells higher), treatment regimen and number of drugs in regimen. *Excluding individuals with a CD4 cell count ≥750 cells/μL at baseline.

In total, 11,255/13,703 (82.1%) individuals had CD4 measurements available after 12±3 months (51.7% on an INSTI, 22.7%, on a PI/b and 25.6% on an NNRTI). The crude proportion of individuals achieving a 25% increase in CD4 count from baseline was lowest for individuals on INSTIs compared to individuals on PI/b or NNRTIs (41.2% [CI 40.0–42.4] vs. 53.0% [51.4–55.3] or 44.6% [42.6–46.4]; p<0.001, respectively; Fig 1). However, after adjustments, individuals on a PI/b or NNRTI had significantly lower odds of achieving a 25% CD4 cell increase, compared to those on an INSTI (0.87 [0.76–0.99], p = 0.04 and 0.80 [0.71–0.91], p <0.001 respectively; Fig 4A).

Of participants with available CD4 counts 8,710 (77.4%) had a CD4 count <750 cells/μL at baseline and could be assessed for achieving a CD4 count ≥750 cells/μL(49.3% on an INSTI, 24.8% on a PI/b and 25.9% on a NNRTI). The crude proportion reaching this level was higher in the INSTI group than in both the PI/b and NNRTI groups (20.9% [19.9–21.9] vs 15.6% [14.3–16.9] and 18.2% [16.9–19.5], p <0.001; Fig 1). After adjustment, compared to individuals on an INSTI, those on a PI/b or an NNRTI had lower odds of achieving a CD4 count ≥750 cells/μL (aOR 0.84 [0.70–1.01], p = 0.06 or 0.62 [0.52–0.72], P <0.001 respectively; Fig 4B), although this did not reach statistical significance for the PI/b group.

### Subgroup analyses

The proportions of individuals with cTO success, on-treatment success, a 25% increase in CD4 cell numbers or reaching a CD4 count of ≥750 cells/μL, stratified into the three pre-specified sub-groups, followed the trends of the overall analyses (S2 Table). Similarly, reasons for cTO failure by treatment status at baseline followed the trends of the overall analyses (see S3 Table and S1 Fig).

In adjusted analyses, achieving cTO success, on-treatment success or a 25% increase in CD4 count did not differ according to ART- and viremia-status at baseline (p>0.05 for all interactions; Figs 2 and 4A), although there was some evidence suggesting that reaching a CD4 count of ≥750 cells/μL differed between strata (interaction p = 0.015; Fig 4B). Among ART-experienced individuals with a baseline VL ≥200, those treated with an NNRTI were significantly more likely to reach ≥750 cells/μL compared to those on INSTIs, in contrast to the results in the overall analysis.

### Sensitivity analyses

**Fig 5:**
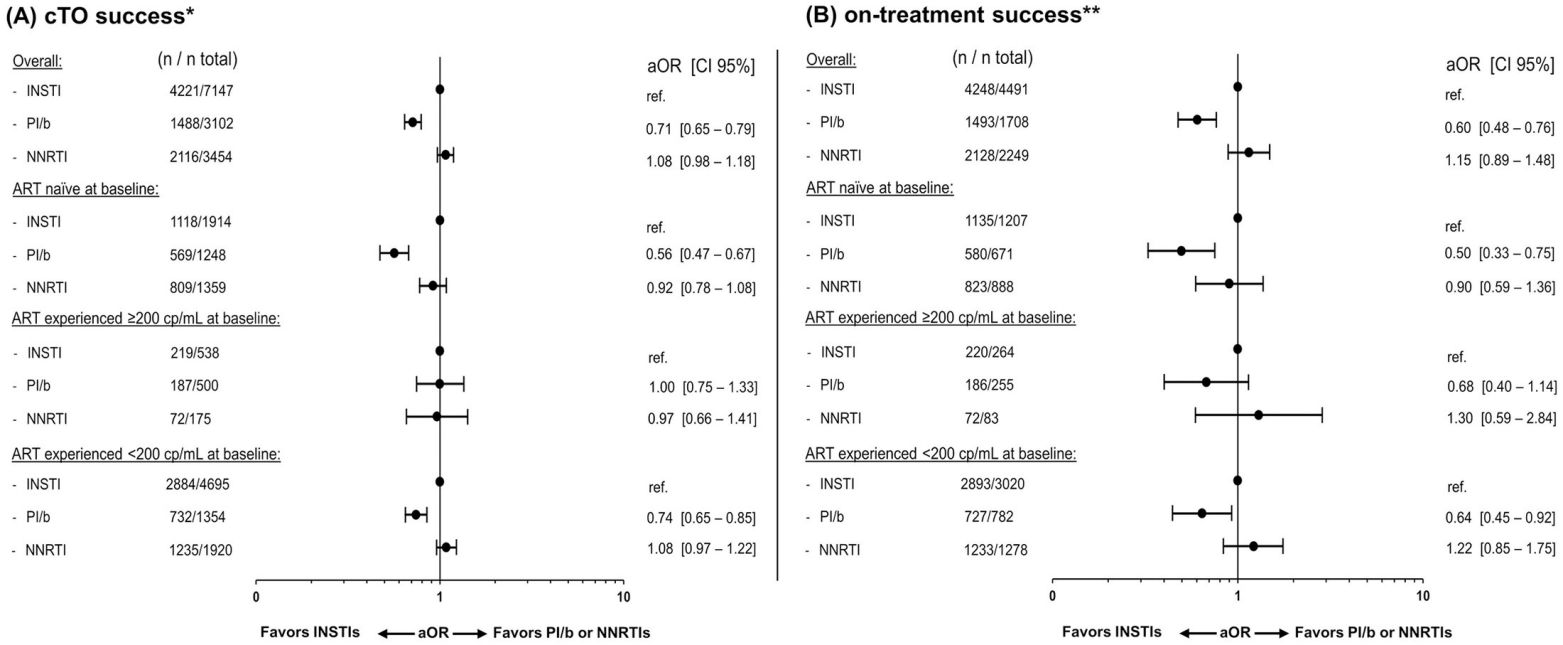
Sensitivity analysis: Adjusted odds ratios (aOR) of cTO and on-treatment analysis with VL cut-off <50 cp/mL at 12±3 months. Multivariable models were adjusted for age (per ten years older), ethnicity, mode of transmission, baseline date (per year later), smoking status, hypertension, diabetes, prior AIDS event- cardiovascular disease, chronic kidney disease, end stage liver disease, non-AIDS-defining malignancies and fractures, HBV and HCV status, viral load (<200 cp/mL, ≥200 cp/mL at baseline), CD4 count (nadir and baseline; both per 100 cells higher), treatment regimen and number of drugs in regimen. * cTO failure was defined as ≥1 of VL ≥50 cp/mL, unknown VL, any ART regimen change, AIDS events, or death. ** Individuals with known VL at 12±3 months without ART regimen change in the period.

When a VL cut-off of <50 cp/mL was used to define cTO (Fig 5A) and on-treatment success (Fig 5B), results were entirely consistent with the primary analysis. Likewise, cTO results were similar when adjusting for cohort rather than comorbidities, or when individuals with unknown VL at 12±3 months were excluded from the cTO definition (S2 Fig).

Excluding either any regimen changes from the cTO definition, or not defining NRTI-backbone changes as regimen changes, both caused cTO success to become slightly less likely for NNRTIs, whereas the relationship between INSTIs and PI/b did not change (see S2 Fig).

**Fig 6:**
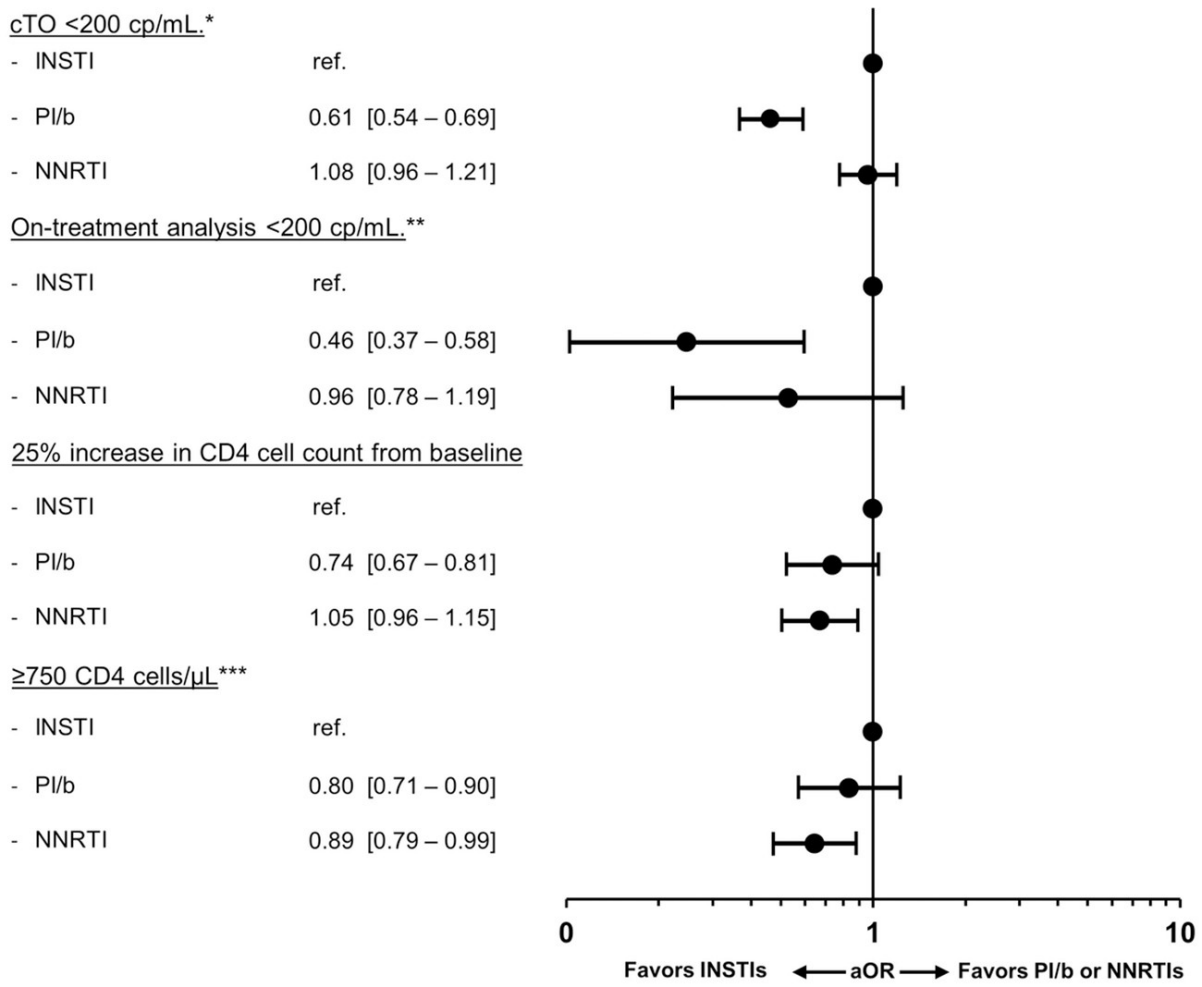
Adjusted odds ratios of cTO success, on-treatment success and favorable immunologic outcomes at 12 ± 3 months for ART-naïve individuals initiating treatment after 16^th^ January 2014. Forest plots showing the aOR of cTO success, on-treatment success, and immunologic response as a 25% increase in CD4 cell counts or reaching a CD4 cell count ≥750 cells/μL. A controlled viral load was defined as <200 cp/mL. Multivariable models were adjusted for age (per ten years older), ethnicity, mode of transmission, baseline date (per year later), baseline smoking status, hypertension, diabetes, HBV and HCV status, prior AIDS event, cardiovascular disease, chronic kidney disease, end stage liver disease, non-AIDS-defining malignancies and prior fractures, viral load (<200 cp/mL, ≥200 cp/mL at baseline), CD4 count (nadir and baseline; both per 100 cells higher, treatment regimen and number of drugs in regimen. *cTO failure was defined as ≥1 either of VL ≥200 cp/mL, unknown VL, ART regimen change, AIDS events, or death. ** Individuals with known VL at 12±3 months without ART regimen change in the period. ***Excluding individuals with ≥750 CD4 cells/μL at baseline.

Analyses from ART-naïve individuals initiating treatment after January 2014, were similar to the primary analyses (Fig 6).

## Discussion

Here we present for the first time real-world data from a large, heterogeneous and geographically diverse multicenter cohort consortium, comparing virologic and immunologic outcomes of treatment with INSTI-containing regimens to contemporary PI/b- and NNRTI-containing regimens. Looking at outcomes after 12 months, using several different definitions for virologic and immunologic outcome, INSTI- and NNRTI-based regimens were, independently of previous treatment and ongoing viremia at treatment start or switch, consistently associated with higher rates of treatment durability and virologic efficacy than PI/b-based regimens. Further, favorable immunologic outcomes were more likely for individuals treated with INSTIs than for individuals treated with NNRTIs and, to a lesser degree, with a PI/b. Here we extend findings from previous RCTs and observational studies [1, 2, 4, 5, 7, 16–19], adding novel evidence favoring INSTI treatment for the general population of PLWH seen in routine clinical care.

### Virologic outcomes

The higher likelihood of cTO success for individuals treated with INSTIs or NNRTIs, compared to individuals treated with a PI/b, was partly due to more frequent regimen changes in the latter group. Though lesser availability of PI/b-containing single-tablet regimes may have contributed to some of these changes, PI/b are well known for having a higher potential for drug-drug interactions and adverse effects, such as gastrointestinal symptoms, than INSTIs and NNRTIs [9].

Previous findings within RESPOND [20] have confirmed that INSTI toxicity only accounts for approximately 5% of the reasons for INSTI discontinuations, similar to findings from the Swiss cohort study [21]. Likewise, a recent, large online-questionnaire examination from the Brazilian Ministry of Health found that only 2.2% of participants experienced self-reported dolutegravir toxicity.

Results from these prior reports, along with our results showing fewer regimen changes on INSTIs, indicate that, although specific toxicities may be overrepresented in individuals treated with specific INSTIs (e.g. neuropsychiatric adverse events with dolutegravir) [20–22], short term INSTI toxicities still appear to be infrequent.

Both cTO and on-treatment analysis revealed a lower likelihood of virologic control for individuals treated with a PI/b compared to an INSTI, as has previously been described [2, 6, 23, 24]. Although we found statistically significant differences in efficacy, all three drug classes individually demonstrated high levels of viral suppression. Further, we found some differences in the crude proportions of AIDS events and deaths between classes over 12 months follow-up. In addition to the higher rates of virologic failure, a larger proportion of individuals in the PI/b group experienced an AIDS defining event during the assessed period. Darunavir/b has a high genetic barrier and was the most frequently prescribed PI/b in this analysis. We are therefore unable to fully exclude the possibility that some of this may be related to confounding by indication. The larger proportion of deaths in the INSTI group could similarly be attributed to confounding by indication, with those in the INSTI group generally being older, more treatment-experienced, and having more comorbidities. Longer term follow-up in large cohort collaborations will be essential to determine whether the relatively modest differences observed here can be explained by adjustment for potential confounding factors or if they will become more pronounced over time.

The proportions of individuals with unknown VL within the 12±3 months time window were relatively high for all three drug classes and without inter-class difference, as also reflected by the consistent results when excluding unknown VL from the cTO. While current recommendations suggest that VL should be measured at least every twelve months for individuals on stable ART, and more frequently following regimen switches [8–11], it is not unreasonable to assume that real-life practice differs on national levels, and more time can pass—in particularly for treatment compliant individuals with suppressed viremia.

As this is an observational study, we cannot with certainty determine whether the lack of VL measurements was a consequence of data not being reported to RESPOND, or of tests not being performed locally within the time period. However, quality assessments are performed as RESPOND data is electronically transferred from all participating sites [14, 25], and therefore it is unlikely that a significant amount of the unknown VLs are due to systematic underreporting to RESPOND.

### Immunologic outcome

We saw only a very small difference in the likelihood of a 25% increase in CD4 count between INSTIs and PI/b, and no difference in the likelihood of achieving ≥750 CD4 cells/μL between the two ARV classes, consistent with prior reports [6, 7]. Similarly to previously findings from large observational studies and RCTs comparing dolutegravir [5, 18] and elvitegravir [4] to efavirenz, we found that individuals treated with NNRTI-containing regimens were less likely to achieve a favorable immunologic response compared to INSTIs, even with rilpivirine added to the NNRTI group in our analysis.

Although the differences found between drug classes were minor, the potential effect on ongoing inflammation and longer-term disease progression is to date not known. Therefore, it will be imperative to observe if these differences will be associated with more frequent adverse outcomes over longer periods of time.

### Subgroup analyses

Although there was some evidence suggesting that reaching a CD4 count ≥750 cells/μL differed slightly according to ART status and level of viremia at baseline, we saw no evidence of a subgroup effect on any of the other outcomes, and the results should be interpreted accordingly with multiple testing in mind. Specifically, for the assessment of outcomes for ART-experienced individuals with VL ≥200 cp/mL at baseline, it should be noted, that due to the comparatively lower resistances barrier of NNRTIs, clinicians may be reluctant to prescribe this drug class to ART-experienced individuals with ongoing viremia, likely reflected in the low number of individuals on NNRTIs in this stratum.

Overall, these findings suggest that INSTIs can safely be used regardless of treatment status and history at treatment initiation, though more studies with comprehensive data on resistance testing is still needed.

Interactions between high VL, low CD4 counts and age with virologic and immunologic outcomes have been investigated in another RESPOND analysis, where no differences were seen between these individuals treated with INSTIs, PI/b and NNRTIs [26].

### Limitations

There are some study limitations to acknowledge. Firstly, our study is observational in nature, and although our analyses included adjustment for a wide range of potential confounders, and several sensitivity and subgroup analyses were performed, residual confounding can never be fully excluded, and our results should be interpreted accordingly. In particular, RESPOND does not systematically collect data on ARV-resistance nor HIV-subtypes, which can affect treatment choice, especially in the strata of ART-experienced individuals with VL ≥200 cp/mL at baseline.

Secondly, as we wanted to ensure adequate power to reliably analyse outcomes and perform predefined subgroup analyses and test for potential interactions, we focused on class effects rather than on individual ARVs. Therefore, it is possible that there may be some intra-class differences not accounted for here. Finally, our analyses were restricted to a 12 months period, and we therefore cannot make any statements on longer-term outcomes. Longer follow-up on individual ARVs is required to allow for individual comparisons, as well as assessment of longer term clinical outcomes including serious non-AIDS clinical events.

## Conclusion

In conclusion, this large, real-world based analysis of a heterogeneous population of PLWH seen in routine clinical care, showed that treatment with INSTI and NNRTI-containing regimes was preferable to PI/b with regard to virologic outcomes, although the potential for residual confounding cannot be fully excluded. Favorable immunologic responses were more likely with INSTI-containing regimens than with NNRTI-containing regimens, and to a lesser degree with PI/b-containing regimens. Crude numbers did not reveal any major differences in the occurrence of AIDS or death. These data supports the use of INSTI treatment and suggest that 12 months efficacy and durability of INSTIs are independent of prior treatment status and on-going viremia.

## Supporting information

S1 Table Baseline demographics and clinical characteristics of individuals stratified by treatment status at baseline. All percentages are column percentages. The proportion of individuals with unknown/missing data were (%): Mode of transmission 6.8; ethnicity 11.8; hepatitis B 16.8; hepatitis C 13.8; BMI 29.5; smoking status 39.0; hypertension 28.9; diabetes 8.5; cardiovascular disease 26.3; chronic kidney disease 19.2. Regions: West Central Europe: Austria, Belgium, France, Germany, Luxembourg, Switzerland; South Europe and Argentina: Argentina, Greece, Israel, Italy, Portugal, Spain; North Europe and Australia: Australia, Denmark, Norway, Sweden, Finland, Iceland, Ireland, Netherlands, United Kingdom; East Central Europe: Bosnia-Herzegovina, Croatia, Czech Republic, Hungary, Poland, Romania, Serbia, Slovenia, Slovakia; Eastern Europe: Belarus, Estonia, Georgia, Latvia, Lithuania, Russia, Ukraine. Hypertension: Defined as use of antihypertensive drugs, systolic blood pressure >140 mmHg and/or diastolic blood pressure >90 mmHg. Diabetes: A clinical diagnosis of diabetes, use of antidiabetic drugs and/or blood glucose measurement ≥11.1 mmol/l or HbA1C ≥48 mmol/mol. HCV Positive: Positive if ever had a positive HCV antibody test, HCV RNA test, HCV genotype, or received HCV treatment prior to baseline. HBV positive: Hepatitis B surface antigen positive. Chronic kidney disease: Defined as confirmed (>3 months apart) eGFR ≤ 60 ml/min/1.73 m2, calculated by the CKD-EPI formula. Cardiovascular disease includes prior myocardial infarction, stroke or invasive cardiovascular procedure. *Among ART-experienced. Abbreviations: 3TC, lamivudine; ABC, abacavir; ART, anti-retroviral therapy; ARV, anti-retroviral drug; BMI, body-mass index; cp, copies of RNA; FTC, emtricitabine; IDU, intravenous drug user; MSM, men who have sex with men; NRTI, nucleos(t)ide reverse transcriptase inhibitor; TDF: tenofovir disoproxil fumarate; TAF: tenofovir alafenamide; VL, viral load. All p-values for comparisons were < 0.001.
(PDF)

S2 Table Numbers and proportions of individuals with cTO success, on-treatment success and immunologic responses at 12 ± 3 months, stratified by treatment status at baseline. *cTO success defined as a VL <200 cp/mL. in individuals without failure (at least one of: VL ≥200 cp/mL, unknown VL, ART regimen change, AIDS events or death). **persons with known VL at 12±3 months without regimen changes (N = INSTI 4513; PI/b 1721; NNRTI 2263). ***persons with known CD4 counts at 12±3 months (N = INSTI 5823; PI/b 2550; NNRTI 2882). ****persons with known CD4 counts at 12±3 months excluding persons with ≥750 CD4 cells/μL at baseline (N = INSTI 4297; PI/b 2160; NNRTI 2253).
(PDF)

S3 Table Reasons for cTO failure* according to treatment status at baseline. *cTO failure was defined as at least one of: VL ≥200 cp/mL, unknown VL, any antiretroviral treatment (ART)-regimen change, AIDS, or death (note that it is possible to fail more than one parameter). ** number of specific reasons for cTO failure. ***persons with known VL at 12±3 months without regimen changes.
(PDF)

S1 Fig Reasons for cTO failure according to treatment status at baseline. *persons with known VL at 12±3 months, without regimen changes. Numbers above each column indicate the total number of the specific reasons for cTO failure overall, and for each of the treatment groups (INSTI, bPI or NNRTI). Numbers in the bars indicate percent of total. The table below the bars shows the numbers individuals with each specific reason for cTO failure by treatment group, stratified by treatment status at baseline. Note that individuals could fail the cTO outcome for more than one reasons.
(TIF)

S2 Fig Forest plots of sensitivity analyses investigating cTO under different conditions. Multivariable models were adjusted for age (per ten years older), ethnicity, mode of transmission, baseline date (per year later), smoking status, hypertension, diabetes, prior AIDS event- cardiovascular disease, chronic kidney disease, end stage liver disease, non-AIDS-defining malignancies and fractures, HBV and HCV status, viral load (<200 cp/mL, ≥200 cp/mL at baseline), CD4 count (nadir and baseline; both per 100 cells higher), treatment regimen and number of drugs in regimen. 1: aOR of cTO success defined as a VL <200 cp/mL. in individuals without failure (at least one of: VL ≥200 cp/mL, unknown VL, cART regimen change, AIDS events or death). 2: aOR of cTO success defined as in 1, at 6±3 months after baseline. 3 aOR of cTO success defined as in 1, restricted to individuals initiating or shifting to one of the study regimens after 16th January 2014. 4 aOR of cTO success defined as in 1, restricted to ART-naïve individuals initiating one of the study regimens after 16^th^ January 2014. 5: aOR of cTO success defined as in 1, model adjusted for cohort instead of comorbidities. 6: aOR of cTO success defined as a VL <200 cp/mL in individuals without failure (at least one of: VL ≥200 cp/mL, unknown VL, AIDS events or death; *excluding ART regime changes from the main cTO outcome*). 7: aOR of cTO success defined as a VL <200 cp/mL in individuals without failure (at least one of: VL ≥200 cp/mL, unknown VL, AIDS events, death or change of 3^rd^ ARV (INSTI, PI/b or NNRTI); *not defining changes in NRTI backbone as an ART regimen change*. 8: aOR of cTO defined as a VL <200 cp/mL in individuals without failure (at least one of: VL ≥200 cp/mL, cART regimen change, AIDS events or death; *excluding unknown VL from main cTO outcome*).
(TIF)

S1 Appendix (PDF)
