# Supplementary material for: Virologic and immunologic outcomes of treatment with integrase inhibitors in a real-world setting: The RESPOND cohort consortium
Source: PLoS One. 2020 Dec 31;15(12):e0243625. doi: 10.1371/journal.pone.0243625 (PMC7774984; doi:10.1371/journal.pone.0243625)
Supplement: S1 Table — All percentages are column percentages. The proportion of individuals with unknown/missing data were (%): Mode of transmission 6.8; ethnicity 11.8; hepatitis B 16.8; hepatitis C 13.8; BMI 29.5; smoking status 39.0; hypertension 28.9; diabetes 8.5; cardiovascular disease 26.3; chronic kidney disease 19.2. Regions: West Central Europe: Austria, Belgium, France, Germany, Luxembourg, Switzerland; South Europe and Argentina: Argentina, Greece, Israel, Italy, Portugal, Spain; North Europe and Australia: Australia, Denmark, Norway, Sweden, Finland, Iceland, Ireland, Netherlands, United Kingdom; East Central Europe: Bosnia-Herzegovina, Croatia, Czech Republic, Hungary, Poland, Romania, Serbia, Slovenia, Slovakia; Eastern Europe: Belarus, Estonia, Georgia, Latvia, Lithuania, Russia, Ukraine. Hypertension: Defined as use of antihypertensive drugs, systolic blood pressure >140 mmHg and/or diastolic blood pressure >90 mmHg. Diabetes: A clinical diagnosis of diabetes, use of antidiabetic drugs and/or blood glucose measurement ≥11.1 mmol/l or HbA1C ≥48 mmol/mol. HCV Positive: Positive if ever had a positive HCV antibody test, HCV RNA test, HCV genotype, or received HCV treatment prior to baseline. HBV positive: Hepatitis B surface antigen positive. Chronic kidney disease: Defined as confirmed (>3 months apart) eGFR ≤ 60 ml/min/1.73 m2, calculated by the CKD-EPI formula. Cardiovascular disease includes prior myocardial infarction, stroke or invasive cardiovascular procedure. *Among ART-experienced. Abbreviations: 3TC, lamivudine; ABC, abacavir; ART, anti-retroviral therapy; ARV, anti-retroviral drug; BMI, body-mass index; cp, copies of RNA; FTC, emtricitabine; IDU, intravenous drug user; MSM, men who have sex with men; NRTI, nucleos(t)ide reverse transcriptase inhibitor; TDF: tenofovir disoproxil fumarate; TAF: tenofovir alafenamide; VL, viral load. All p-values for comparisons were < 0.001. (PDF) [file pone.0243625.s001.pdf]

S1 Table. Baseline demographics and clinical characteristics of individuals stratified by treatment status at baseline.

		All		ART-Naïve		ART-Experienced VL ≥200 cp/mL at baseline		ART-Experienced VL <200 cp/mL at baseline	
		n	%	n	%	n	%	n	%
		**13703**	**100.0**	**7147**	**52.2**	**3102**	**22.6**	**3454**	**25.2**
Sex	Female	10313	(75.3)	3723	(82.3)	5839	(73.3)	751	(61.9)
	Male	3390	(24.7)	798	(17.7)	2130	(26.7)	462	(38.1)
Ethnicity	White	9625	(70.2)	3166	(70.0)	5718	(71.8)	741	(61.1)
	Other	2162	(15.8)	670	(14.8)	1180	(14.8)	312	(25.7)
	Unknown	1916	(14.0)	685	(15.2)	1071	(13.4)	160	(13.2)
Region	South Europe and Argentina	3825	(27.9)	2039	(45.1)	1585	(19.9)	201	(16.6)
	West Central Europe	7364	(53.7)	1923	(42.5)	4708	(59.1)	733	(60.4)
	North Europe and Australia	1489	(10.9)	302	(6.7)	1055	(13.2)	132	(10.9)
	Central East Europe	623	(4.5)	62	(1.4)	480	(6.0)	81	(6.7)
	East Europe	402	(2.9)	195	(4.3)	141	(1.8)	66	(5.4)
Route of HIV acquisition	MSM	6322	(46.1)	2604	(57.6)	3372	(42.3)	346	(28.5)
	IDU	1768	(12.9)	267	(5.9)	1275	(16.0)	226	(18.6)
	Heterosexual	4626	(33.8)	1368	(30.3)	2722	(34.2)	536	(44.2)
	Other/Unknown	987	(7.2)	282	(6.2)	600	(7.5)	105	(8.7)
ARV anchor drug	INSTI	7147	(52.2)	1914	(42.3)	538	(44.4)	4695	(58.9)
	PI/b	3102	(22.6)	1248	(27.6)	500	(41.2)	1354	(17.0)
	NNRTI	3454	(25.2)	1359	(30.1)	175	(14.4)	1920	(24.1)
NRTI Backbone	TDF/FTC	8158	(59.5)	3314	(73.3)	4103	(51.5)	741	(61.1)
	TAF/FTC	1116	(8.1)	307	(6.8)	762	(9.6)	47	(3.9)
	ABC/3TC	3720	(27.1)	826	(18.3)	2604	(32.7)	290	(23.9)
	Other	709	(5.2)	74	(1.6)	500	(6.3)	135	(11.1)
Prior AIDS	Yes	2741	(20.0)	373	(8.3)	1991	(25.0)	377	(31.1)
Hepatitis B	Positive	633	(4.6)	114	(2.5)	459	(5.8)	60	(4.9)
Hepatitis C	Positive	2731	(19.9)	382	(8.4)	2042	(25.6)	307	(25.3)
BMI (kg/m²)	≤18	332	(2.4)	102	(2.3)	187	(2.3)	43	(3.5)
	18-25	6143	(44.8)	1830	(40.5)	3773	(47.3)	540	(44.5)
	25-30	2716	(19.8)	640	(14.2)	1879	(23.6)	197	(16.2)
	>30	877	(6.4)	153	(3.4)	634	(8.0)	90	(7.4)
Smoking	Current	4241	(30.9)	1192	(26.4)	2622	(32.9)	427	(35.2)
Hypertension	Yes	3402	(24.8)	603	(13.3)	2512	(31.5)	287	(23.7)
Diabetes	Yes	731	(5.3)	77	(1.7)	590	(7.4)	64	(5.3)
Cardiovascular disease	Yes	386	(2.8)	16	(0.4)	342	(4.3)	28	(2.3)
Chronic kidney disease	Yes	400	(2.9)	4	(0.1)	363	(4.6)	33	(2.7)

	All		ART-Naïve		ART-Experienced VL ≥200 cp/mL at baseline		ART-Experienced VL <200 cp/mL at baseline	
	**n**	**%**	**n**	**%**	**n**	**%**	**n**	**%**
	**13703**	**100.0**	**7147**	**52.2**	**3102**	**22.6**	**3454**	**25.2**
**Continuous variables**	**Median**	**(IQR)**	**Median**	**(IQR)**	**Median**	**(IQR)**	**Median**	**(IQR)**
Age (years)	46	[37-53]	38	[31-47]	49	[42-55]	44	[37-51]
Baseline CD4 (cells/µL)	510	[328-724]	360	[200-510]	629	[460-828]	321	[151-509]
Nadir CD4 (cells/µL)	228	[105-358]	340	[191-468]	195	[88-290]	155	[51-283]
ARV drugs previous taken*(n)	6	[3-8]	-	-	6	[4-8]	5	[3-7]
Years since initiation of first ARV* (years)	11	[6-17]	-	-	11	[6-17]	9	[4-14]
Baseline date (mm/yy)	10/14	[07/13 – 08/16]	06/14	[10/14-04/16]	07/13	[07/12-09/14]	06/13	[03/12-08/14]

All percentages are column percentages

The proportion of individuals with unknown/missing data were (%): Mode of transmission 6.8; ethnicity 11.8; hepatitis B 16.8; hepatitis C 13.8; BMI 29.5; smoking status 39.0; hypertension 28.9; diabetes 8.5; cardiovascular disease 26.3; chronic kidney disease 19.2.

Regions: West Central Europe: Austria, Belgium, France, Germany, Luxembourg, Switzerland; South Europe and Argentina: Argentina, Greece, Israel, Italy, Portugal, Spain; North Europe and Australia: Australia, Denmark, Norway, Sweden, Finland, Iceland, Ireland, Netherlands, United Kingdom; East Central Europe: Bosnia-Herzegovina, Croatia, Czech Republic, Hungary, Poland, Romania, Serbia, Slovenia, Slovakia; Eastern Europe: Belarus, Estonia, Georgia, Latvia, Lithuania, Russia, Ukraine .

Hypertension: Defined as use of antihypertensive drugs, systolic blood pressure >140 mmHg and/or diastolic blood pressure >90 mmHg.

Diabetes: A clinical diagnosis of diabetes, use of antidiabetic drugs and/or blood glucose measurement ≥11.1 mmol/l or HbA1C ≥48 mmol/mol.

HCV Positive: Positive if ever had a positive HCV antibody test, HCV RNA test, HCV genotype, or received HCV treatment prior to baseline. HBV positive: Hepatitis B surface antigen positive

Chronic kidney disease: Defined as confirmed (>3 months apart) eGFR ≤ 60 ml/min/1.73 m2, calculated by the CKD-EPI formula.

Cardiovascular disease includes prior myocardial infarction, stroke or invasive cardiovascular procedure.

*Among ART-experienced

Abbreviations: 3TC, lamivudine; ABC, abacavir; ART, anti-retroviral therapy; ARV, anti-retroviral drug; BMI, body-mass index; cp, copies of RNA; FTC, emtricitabine; IDU, intravenous drug user; MSM, men who have sex with men; NRTI, nucleos(t)ide reverse transcriptase inhibitor; TDF: tenofovir disoproxil fumarate; TAF: tenofovir alafenamide; VL, viral load.

All p-values for comparisons were < 0.001
