# Supplementary material for: Virologic and immunologic outcomes of treatment with integrase inhibitors in a real-world setting: The RESPOND cohort consortium
Source: PLoS One. 2020 Dec 31;15(12):e0243625. doi: 10.1371/journal.pone.0243625 (PMC7774984; doi:10.1371/journal.pone.0243625)
Supplement: S2 Table — *cTO success defined as a VL <200 cp/mL. in individuals without failure (at least one of: VL ≥200 cp/mL, unknown VL, ART regimen change, AIDS events or death). **persons with known VL at 12±3 months without regimen changes (N = INSTI 4513; PI/b 1721; NNRTI 2263). ***persons with known CD4 counts at 12±3 months (N = INSTI 5823; PI/b 2550; NNRTI 2882). ****persons with known CD4 counts at 12±3 months excluding persons with ≥750 CD4 cells/μL at baseline (N = INSTI 4297; PI/b 2160; NNRTI 2253). (PDF) [file pone.0243625.s002.pdf]

S2 Table. Numbers and proportions of individuals with cTO success, on-treatment success and immunologic responses at 12 ± 3 months, stratified by treatment status at baseline

Outcomes	Treatment status at baseline	All				INSTI				PI/b				NNRTI				p-value
		n	total	(%)	95% CI	n	total	(%)	95% CI	n	Total	(%)	95% CI	n	total)	(%)	95% CI	
cTO <200 cp/mL*	All	8215	13703	(60.0)	[59.1 - 60.8]	4398	7147	(61.5)	[60.4 - 62.7]	1616	3102	(52.1)	[50.3 - 53.9]	2201	3454	(63.7)	[62.1 - 65.3]	<0.001
	ART-Naïve	2642	4521	(58.4)	[57.0 - 59.9]	1169	1914	(61.1)	[58.9 - 63.3]	622	1248	(49.8)	[47.1 - 52.6]	851	1359	(62.6)	[60.0 - 65.2]	<0.001
	ART-experienced, VL ≥200 cp/mL at baseline	528	1213	(43.5)	[40.7 - 46.3]	240	538	(44.6)	[40.4 - 48.8]	216	500	(43.2)	[38.9 - 47.5]	72	175	(41.1)	[33.9 - 48.4]	0.71
	ART-experienced, VL <200 cp/mL at baseline	5045	7969	(63.3)	[62.2 - 64.4]	2989	4695	(63.7)	[62.3 - 65.0]	778	1354	(57.5)	[54.8 - 60.1]	1278	1920	(66.6)	[64.5 - 68.7]	<0.001
On-treatment analysis <200 cp/mL**	All	8273	8497	(97.4)	[97.0 - 97.7]	4431	4513	(98.2)	[97.8 - 98.6]	1628	1721	(94.6)	[93.5 - 95.7]	2214	2263	(97.8)	[97.2 - 98.4]	<0.001
	ART-Naïve	2693	2780	(96.9)	[96.2 - 97.5]	1189	1211	(98.2)	[97.4 - 98.9]	638	674	(94.7)	[93.0 - 96.4]	866	895	(96.8)	[95.6 - 97.9]	<0.001
	ART-experienced, VL ≥200cp/mL at baseline	532	604	(88.1)	[85.5 - 90.7]	242	265	(91.3)	[87.9 - 94.7]	218	256	(85.2)	[80.8 - 89.5]	72	83	(86.7)	[79.5 - 94.0]	0.08
	ART-experienced, VL <200 cp/mL at baseline	5048	5113	(98.7)	[98.4 - 99.0]	3000	3037	(98.8)	[98.4 - 99.2]	772	791	(97.6)	[96.5 - 98.7]	1276	1285	(99.3)	[98.8 - 99.8]	0.003
≥25 % CD4 increase in count from baseline ***	All	5046	11255	(44.8)	[43.9 - 45.8]	2399	5823	(41.2)	[40.0 - 42.5]	1361	2550	(53.4)	[51.4 - 55.3]	1286	2882	(44.6)	[42.6 - 46.4]	<0.001
	ART-Naïve	3072	3815	(67.9)	[66.5 - 69.4]	1315	1616	(81.4)	[78.9 - 83.8]	875	1054	(83.0)	[80.8 - 85.3]	882	1145	(77.0)	[74.6 - 79.5]	<0.001
	ART-experienced, VL ≥200 cp/mL at baseline	531	934	(43.8)	[40.6 - 47.0]	228	407	(56.0)	[50.5 - 61.5]	222	389	(57.1)	[52.2 - 62.0]	81	138	(58.7)	[50.5 - 66.9]	0.3137
	ART-experienced, VL <200 cp/mL at baseline	1443	6506	(18.1)	[17.2 - 19.0]	856	3800	(22.5)	[21.2 - 23.9]	264	1107	(23.8)	[21.3 - 26.4]	323	1599	(20.2)	[18.2 - 22.2]	0.06
≥750 CD4 cells/µL ****	All	1644	8710	(18.9)	[18.1 - 19.7]	897	4297	(20.9)	[19.5 - 22.2]	337	2160	(15.6)	[14.1 - 17.1]	410	2253	(18.2)	[16.6 - 19.8]	<0.001
	ART-Naïve	867	3556	(24.4)	[23.0 - 25.8]	430	1469	(29.3)	[28.0 - 30.6]	203	1016	(20.0)	[17.5 - 22.4]	234	1071	(21.8)	[19.4 - 24.3]	<0.001
	ART-experienced, VL ≥200 cp/mL at baseline	109	865	(12.6)	[10.4 - 14.8]	45	369	(12.2)	[9.3 - 15.1]	34	367	(9.3)	[6.3 - 12.2]	30	129	(23.3)	[16.0 - 30.5]	<0.001
	ART-experienced, VL <200 cp/mL at baseline	668	4289	(15.6)	[14.5 - 16.7]	422	2459	(17.2)	[15.0 - 19.3]	100	777	(12.9)	[10.5 - 15.2]	146	1053	(13.9)	[11.8 - 16.0]	0.003

**cTO success defined as a VL <200 cp/mL. in individuals without failure (at least one of: VL ≥200 cp/mL, unknown VL, cART regimen changes, AIDS events or death)

**persons with known VL at 12±3 months. without regimen changes (N =INSTI 4513; PI/b 1721; NNRTI 2263)

***persons with known CD4 counts at 12±3 months. (N =INSTI 5823; PI/b 2550; NNRTI 2882)

**** persons with known CD4 counts at 12±3 months. excluding persons with ≥750 CD4 cells/µL at baseline (N =INSTI 4297; PI/b 2160; NNRTI 2253)
