# Supplementary material for: Virologic and immunologic outcomes of treatment with integrase inhibitors in a real-world setting: The RESPOND cohort consortium
Source: PLoS One. 2020 Dec 31;15(12):e0243625. doi: 10.1371/journal.pone.0243625 (PMC7774984; doi:10.1371/journal.pone.0243625)
Supplement: S3 Table — *cTO failure was defined as at least one of: VL ≥200 cp/mL, unknown VL, any antiretroviral treatment (ART)-regimen change, AIDS, or death (note that it is possible to fail more than one parameter). ** number of specific reasons for cTO failure. ***persons with known VL at 12±3 months without regimen changes. (PDF) [file pone.0243625.s003.pdf]

S3 Table. Reasons for cTO failure* according to treatment status at baseline

Reason for cTO failure	Treatment status at baseline	All				INSTI				PI/b				NNRTI				p-value
		n**	total	(%)	95% CI	n**	total	(%)	95% CI	n**	Total	(%)	95% CI	n**	total)	(%)	95% CI	
Viral failure***	All	224	8497	(2.6)	[2.3 - 3.0]	82	4513	(1.8)	[1.4 - 2.2]	93	1721	(5.4)	[4.3 - 6.5]	49	2263	(2.2)	[1.6 - 2.8]	<0.0001
	ART-Naïve	87	2780	(3.1)	[2.5 - 3.8]	22	1211	(1.8)	[1.1 - 2.6]	36	674	(5.3)	[3.6 - 7.0]	29	895	(3.2)	[2.1 - 4.4]	<0.0001
	ART-experienced, VL ≥200 cp/mL at baseline	72	604	(11.9)	[9.3 - 14.5]	23	265	(8.7)	[5.3 - 12.1]	38	256	(14.8)	[10.5 - 19.2]	11	83	(13.3)	[6.0 - 20.5]	0.087
	ART-experienced, VL <200 cp/mL at baseline	65	5113	(1.3)	[1.0 - 1.6]	37	3037	(1.2)	[0.8 - 1.6]	19	791	(2.4)	[1.3 - 3.5]	9	1285	(0.7)	[0.2 - 1.2]	0.0032
Unknown VL	All	2278	13703	(16.6)	[16.0 - 17.2]	1153	7147	(16.1)	[15.3 - 17.0]	537	3102	(17.3)	[16.0 - 18.6]	588	3454	(17.0)	[15.8 - 18.3]	0.26
	ART-Naïve	699	4521	(15.5)	[14.4 - 16.5]	274	1914	(14.3)	[12.7 - 15.9]	197	1248	(15.8)	[13.8 - 17.8]	228	1359	(16.8)	[14.8 - 18.8]	0.14
	ART-experienced, VL ≥200 cp/mL at baseline	295	1213	(24.3)	[21.9 - 26.7]	133	538	(24.7)	[21.1 - 28.4]	119	500	(23.8)	[20.1 - 27.5]	43	175	(24.6)	[18.2 - 30.9]	0.94
	ART-experienced, VL <200 cp/mL at baseline	1284	7969	(16.1)	[15.3 - 16.9]	746	4695	(15.9)	[14.8 - 16.9]	221	1354	(16.3)	[14.4 - 18.3]	317	1920	(16.5)	[14.8 - 18.2]	0.80
Regimen changes	All	3449	13703	(25.2)	[24.4 - 25.9]	1741	7147	(24.4)	[23.4 - 25.4]	981	3102	(31.6)	[30.0 - 33.3]	727	3454	(21.0)	[19.7 - 22.4]	<0.0001
	ART-Naïve	1198	4521	(26.5)	[25.2 - 27.8]	490	1914	(25.6)	[23.6 - 27.6]	424	1248	(34.0)	[31.3 - 36.6]	284	1359	(20.9)	[18.7 - 23.1]	<0.0001
	ART-experienced, VL ≥200 cp/mL at baseline	420	1213	(34.6)	[31.9 - 37.3]	182	538	(33.8)	[29.8 - 37.8]	176	500	(35.2)	[31.0 - 39.4]	62	175	(35.4)	[28.3 - 42.5]	0.87
	ART-experienced, VL <200 cp/mL at baseline	1831	7969	(23.0)	[22.1 - 23.9]	1069	4695	(22.8)	[21.6 - 24.0]	381	1354	(28.1)	[25.7 - 30.5]	381	1920	(19.8)	[18.1 - 21.6]	<0.0001
AIDS	All	201	13703	(1.5)	[1.3 - 1.7]	90	7147	(1.3)	[1.0 - 1.5]	76	3102	(2.5)	[1.9 - 3.0]	35	3454	(1.0)	[0.7 - 1.3]	<0.0001
	ART-Naïve	140	4521	(3.1)	[2.6 - 3.6]	63	1914	(3.3)	[2.5 - 4.1]	48	1248	(3.8)	[2.8 - 4.9]	29	1359	(2.1)	[1.4 - 2.9]	0.034
	ART-experienced, VL ≥200 cp/mL at baseline	37	1213	(3.1)	[2.1 - 4.0]	15	538	(2.8)	[1.4 - 4.2]	18	500	(3.6)	[2.0- 5.2]	4	175	(2.3)	[0.1 - 4.5]	0.61
	ART-experienced, VL <200 cp/mL at baseline	24	7969	(0.3)	[0.2 - 0.4]	12	4695	(0.3)	[0.1 - 0.4]	10	1354	(0.7)	[0.3 - 1.2]	2	1920	(0.1)	[0.0 - 0.2]	0.0033
Death	All	108	13703	(0.8)	[0.6 - 0.9]	75	7147	(1.0)	[0.8 - 1.3]	25	3102	(0.8)	[0.5 - 1.1]	8	3454	(0.2)	[0.1 - 0.4]	<0.0001
	ART-Naïve	31	4521	(0.7)	[0.4 - 0.9]	17	1914	(0.9)	[0.5 - 1.3]	8	1248	(0.6)	[0.2 - 1.1]	6	1359	(0.4)	[0.1 - 0.8]	0.30
	ART-experienced, VL ≥200cp/mL at baseline	27	1213	(2.2)	[1.4 - 3.1]	17	538	(3.2)	[1.7 - 4.6]	10	500	(2.0)	[0.8 - 3.2]	0	175	(0.0)	[0.0 - 0.0]	0.044
	ART-experienced, VL <200 cp/mL at baseline	50	7969	(0.6)	[0.5 - 0.8]	41	4695	(0.9)	[0.6 - 1.1]	7	1354	(0.5)	[0.1 - 0.9]	2	1920	(0.1)	[0.0 - 0.2]	0.0013

*cTO failure was defined as at least one of: VL ≥200 cp/mL., unknown VL., any antiretroviral treatment (ART)-regimen change., AIDS, or death (note that it is possible to fail more than one parameter)
** number of specific reasons for cTO failure
***persons with known VL at 12±3 months. without regimen changes
