# Supplementary figures and images for: Virologic and immunologic outcomes of treatment with integrase inhibitors in a real-world setting: The RESPOND cohort consortium
Source: PLoS One. 2020 Dec 31;15(12):e0243625. doi: 10.1371/journal.pone.0243625 (PMC7774984; doi:10.1371/journal.pone.0243625)

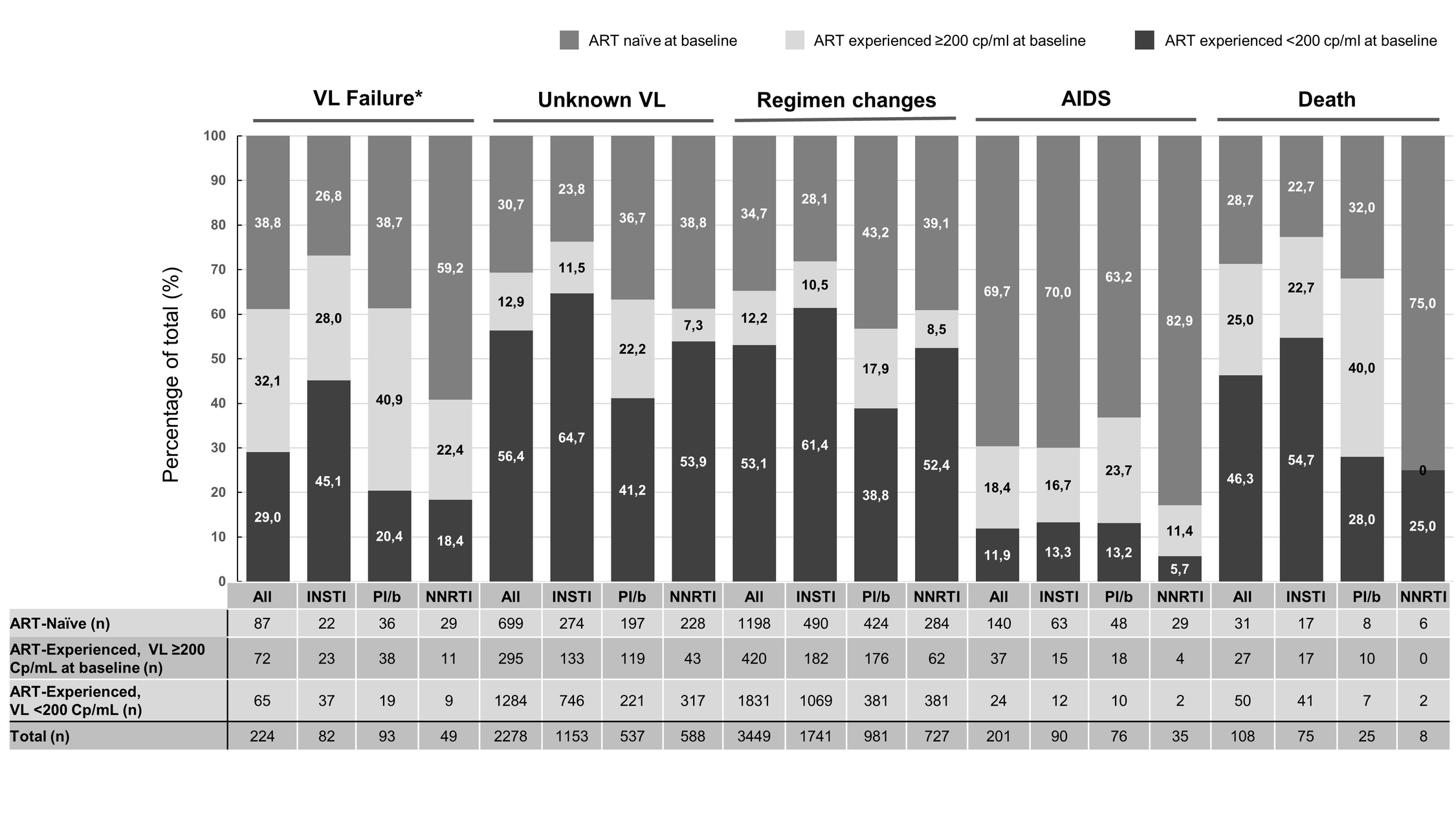

Supplement: S1 Fig — *persons with known VL at 12±3 months, without regimen changes. Numbers above each column indicate the total number of the specific reasons for cTO failure overall, and for each of the treatment groups (INSTI, bPI or NNRTI). Numbers in the bars indicate percent of total. The table below the bars shows the numbers individuals with each specific reason for cTO failure by treatment group, stratified by treatment status at baseline. Note that individuals could fail the cTO outcome for more than one reasons. (TIF) [file pone.0243625.s004.tif]

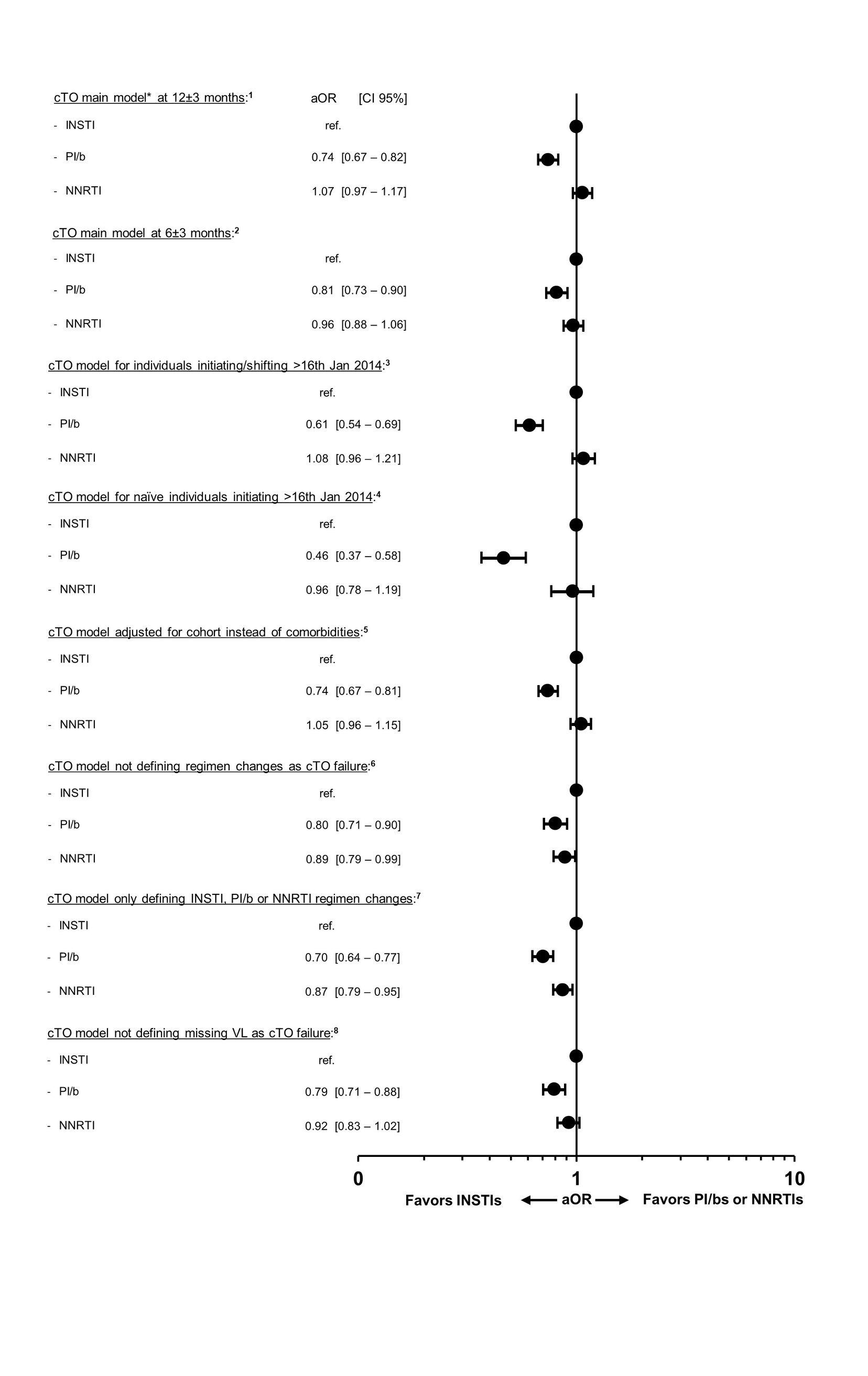

Supplement: S2 Fig — Multivariable models were adjusted for age (per ten years older), ethnicity, mode of transmission, baseline date (per year later), smoking status, hypertension, diabetes, prior AIDS event- cardiovascular disease, chronic kidney disease, end stage liver disease, non-AIDS-defining malignancies and fractures, HBV and HCV status, viral load (<200 cp/mL, ≥200 cp/mL at baseline), CD4 count (nadir and baseline; both per 100 cells higher), treatment regimen and number of drugs in regimen. 1: aOR of cTO success defined as a VL <200 cp/mL. in individuals without failure (at least one of: VL ≥200 cp/mL, unknown VL, cART regimen change, AIDS events or death). 2: aOR of cTO success defined as in 1, at 6±3 months after baseline. 3 aOR of cTO success defined as in 1, restricted to individuals initiating or shifting to one of the study regimens after 16th January 2014. 4 aOR of cTO success defined as in 1, restricted to ART-naïve individuals initiating one of the study regimens after 16th January 2014. 5: aOR of cTO success defined as in 1, model adjusted for cohort instead of comorbidities. 6: aOR of cTO success defined as a VL <200 cp/mL in individuals without failure (at least one of: VL ≥200 cp/mL, unknown VL, AIDS events or death; excluding ART regime changes from the main cTO outcome). 7: aOR of cTO success defined as a VL <200 cp/mL in individuals without failure (at least one of: VL ≥200 cp/mL, unknown VL, AIDS events, death or change of 3rd ARV (INSTI, PI/b or NNRTI); not defining changes in NRTI backbone as an ART regimen change. 8: aOR of cTO defined as a VL <200 cp/mL in individuals without failure (at least one of: VL ≥200 cp/mL, cART regimen change, AIDS events or death; excluding unknown VL from main cTO outcome). (TIF) [file pone.0243625.s005.tif]
