# Supplementary material for: Virologic and immunologic outcomes of treatment with integrase inhibitors in a real-world setting: The RESPOND cohort consortium
Source: PLoS One. 2020 Dec 31;15(12):e0243625. doi: 10.1371/journal.pone.0243625 (PMC7774984; doi:10.1371/journal.pone.0243625)
Supplement: S1 Appendix — (PDF) [file pone.0243625.s006.pdf]

Appendix:

RESPOND inclusion criteria
(https://chip.dk/Portals/0/files/RESPOND/RESPOND%20governance%20and%20procedures_v6_2019SEP30.pdf?ver=2019-10-02-144419-230) :

1. Persons who started an INSTI before 1/1/2012 are excluded from RESPOND

2. Persons aged < 18 at baseline are excluded from RESPOND

3. Persons are included in the INSTI module if have started an INSTI after the later of 1/1/2012 and local cohort enrolment i.e., during prospective follow-up in the cohort and after 1/1/2012) and they have a CD4 and VL measurement in the 12 months prior to starting INSTI or within 3 months after starting INSTI.

4. Persons not treated with an INSTI can be included, providing they have a CD4 and VL measurement within the 12 months prior to baseline or within 3 months after baseline (here, the latest of 1/1/2012 or local cohort enrolment). Persons do not need to have had started ART to be included.

5. Persons lost to follow-up or who died before RESPOND enrolment (01/12/2019) should therefore still be included, provided they satisfy the other inclusion criterion.

6. All relevant data should be included in RESPOND, including data from before RESPOND enrolment and baseline, whenever this is available. This includes all lab data and all antiretroviral therapy (ART) use

7. All clinical events occurring after baseline should be reported with a date of the even
